# Gut Microbiome and the Role of Metabolites in the Study of Graves’ Disease

**DOI:** 10.3389/fmolb.2022.841223

**Published:** 2022-02-16

**Authors:** Haihua Liu, Huiying Liu, Chang Liu, Mengxue Shang, Tianfu Wei, Peiyuan Yin

**Affiliations:** ^1^ Clinical Laboratory of Integrative Medicine, First Affiliated Hospital of Dalian Medical University, Dalian, China; ^2^ Institute of Integrative Medicine, Dalian Medical University, Dalian, China

**Keywords:** graves’ disease (GD), metabol(n)omics, gut microbiome, autoimmunity, metabolites

## Abstract

Graves’ disease (GD) is an autoimmune thyroid disease (AITD), which is one of the most common organ-specific autoimmune disorders with an increasing prevalence worldwide. But the etiology of GD is still unclear. A growing number of studies show correlations between gut microbiota and GD. The dysbiosis of gut microbiota may be the reason for the development of GD by modulating the immune system. Metabolites act as mediators or modulators between gut microbiota and thyroid. The purpose of this review is to summarize the correlations between gut microbiota, microbial metabolites and GD. Challenges in the future study are also discussed. The combination of microbiome and metabolome may provide new insight for the study and put forward the diagnosis, treatment, prevention of GD in the future.

## Introduction

Autoimmune thyroid disease (AITD) are common organ-specific autoimmune disorders with an increasing prevalence worldwide, which involves Hashimoto thyroiditis (HT) and GD (Moshkelgosha et al., 2021). GD is caused by the autoantibodies of the thyrotropin receptor (TSHR), which leads to thyroid hyperplasia and hyperthyroidism (Bahn, 2003; Ishaq et al., 2018; Shi et al., 2019a; Moshkelgosha et al., 2021). Hyperthyroidism, fatigue, weight loss, tachycardia, and heat intolerance are common symptoms of GD. Approximately 50% of patients may develop Graves’ ophthalmopathy (GO), leading to eyelid retractions and exophthalmos (Byeon et al., 2018; Yan et al., 2020). GD is the most common cause of 60–80% of hyperthyroidism and influence about 0.5% of the general population (Cooper and Stroehla, 2003; Smith and Hegedüs, 2016; Ejtahed et al., 2020). It frequently occurs in the population between 30 and 50 years old. Resemble in other autoimmune diseases, the incidence of GD is higher in women than men, the ratio of about 5/1 (Cooper and Stroehla, 2003; Ji et al., 20188; Nyström et al., 2013; Menconi et al., 2014). The risk factors of GD include genetic predisposition, environmental factors, immune factors (Covelli and Ludgate, 2017).

Hyperthyroidism is a common disease that is difficult to cure completely. Although modern medicine has brought great changes to the prevention, diagnosis, and treatment of autoimmune diseases, the etiology and pathogenesis of these diseases have not been fully illuminated. Abnormal thyroid-related indices often occur repeatedly during clinical treatment (Yang et al., 2019). Furthermore, although current treatment methods for GD can achieve a good effect, clinicians still have some concerns about the choice of treatment for safety reasons (Heyma et al., 1986; Yang et al., 2019). At present, a large number of studies have proved the relationship between intestinal microorganisms and autoimmune diseases, including Type 1 diabetes (Gianchecchi and Fierabracci, 2017; Mullaney et al., 2018), inflammatory bowel disease (Ni et al., 2017; Cao, 2018), systemic lupus erythematosus (Corrêa et al., 2017), rheumatoid arthritis (Sato et al., 2017; Teng et al., 2017; Jubair et al., 2018; Picchianti-Diamanti et al., 2018) and autoimmune thyroid disease (Zhou et al., 2014). Metabolites are also considered as important mediators or modulators between gut microbiota and the thyroid. Therefore, metabolomics investigations may provide a new inside view of GD’s study.

In this review, we explore the inside relationships between gut microbiota, microbiota-related metabolites and GD, and propose new ideas for prevention, diagnosis, and treatment of GD.

### Brife Knowledge of Gut Microbiota

The human body is a superorganism due to the residence of trillions of prokaryotes symbiosis. Approximately 66% of the total bacteria are mainly live in the gut. Gut microbiota includes more than one thousand known species of bacteria with at least three million genes (Hehemann et al., 2010; Relman, 2012; Docimo et al., 2020). Apart from absorbing nutrients from the human body that they depend on for survival, intestinal flora also provides beneficial or harmful metabolites to the human body through their metabolic process (Turnbaugh and Gordon, 2009; Relman, 2012). These microflorae participate in the body’s energy metabolism through various mechanisms, affecting the conversion of food to energy in the host, and play an essential role in the healthy state of the host (Lozupone et al., 2012; Sommer et al., 2017). When the human body is healthy, microorganisms and various organs and tissues depend on each other and act on either to form a microecological balance and jointly maintain the body’s health. If the microecological balance is disturbed, it may lead to disease (Sekirov et al., 2010). Therefore, the intestinal flora is considered an “organ” with multiple regulatory functions, which greatly impacts people’s health. Understanding the symbiotic relationship between microorganisms and the human body is of great significance for people to understand their health and the occurrence and development of disease (Turnbaugh and Gordon, 2009; Relman, 2012; Schmidt et al., 2018).

The technological breakthroughs in the microbiome boost the research of gut microbiota. The method of bacterial culture is a restriction of traditional bacterial research. The intestinal flora is cultured with various mediums, and the number of bacterial colonies is measured by dilution and colony count (Lagier et al., 2018). This method is sensitive but is constrained. More than 85% of the bacteria in the human intestine are anaerobic bacteria, which is difficult to cultivate in the culture medium (Lagier et al., 2015). Recently, the newly established strategy of culturomics enables the culture of microbiota that cannot be cultured before. These new methods initiate the rebirth of culture in microbiology (Kaeberlein et al., 2002; Nichols et al., 2010; Lagier et al., 2018). The development of new techniques has made it possible to study unknown gut flora.16Sr RNA high-throughput sequencing and metagenomics are commonly used methods for detecting gut microbiota. 16Sr RNA sequencing mainly studies the species composition, the evolutionary relationships among species and the diversity of communities (Laudadio et al., 2018). On the basis of 16Sr RNA sequencing analysis, metagenomic sequencing can also carry out in-depth research on gene and function, and its detection depth can reach the level of species (Wang et al., 2015; Laudadio et al., 2018; Shakya et al., 2019).

With the increasing understanding of the metabolic function of intestinal flora, the narrow sense that host metabolism is regulated by its genes is gradually expanded to co-metabolic regulation of host-symbiotic intestinal bacteria. These metabolites are often from tryptophan metabolic pathways, tyrosine and phenylalanine metabolic pathways, glucose and fatty acid metabolic pathways, classified into indoles, phenols, amino acids, peptides, etc. (Zheng et al., 2011; Van Treuren and Dodd, 2020; Fan and Pedersen, 2021). Microbiome dysbiosis is associated with various diseases, asthma, allergies, inflammatory bowel disease (Arrieta et al., 2015; Bunyavanich et al., 2016; Nishino et al., 2018), autism spectrum disorder (ASD) (Needham et al., 2021), diabetes (Giongo et al., 2011), irritable bowel syndrome (IBS) (Mars et al., 2020), obesity (Schwiertz et al., 2010a), cardiovascular disease (Jie et al., 2017), chronic kidney disease (Sircana et al., 2019). Under different disease states, the species abundance of intestinal flora and its related metabolites have various characteristics. Some studies have found that in patients with IBS, the key findings include an increase in Firmicutes to Bacteroidetes ratio (Krogius-Kurikka et al., 2009; Rajilić-Stojanović et al., 2011; Jeffery et al., 2012l; Mars et al., 2020), a decrease in Bifidobacteria and Lactobacilli (Malinen et al., 2005; Kerckhoffs et al., 2009), and an increase in Ruminococcus and Streptococci species (Kassinen et al., 2007; Rajilić-Stojanović et al., 2011; Saulnier et al., 2011; Hong and Rhee, 2014). A more coincident finding has been decreased alpha diversity. ASD showed lower levels of phylum Firmicutes and a higher abundance of Bacteroidetes (Mangiola et al., 2016; Fattorusso et al., 2019; Sharon et al., 2019). Kang and others observed significant ASD-related behavioral changes in mice with fecal microbiota transplantation (FMT) from ASD (Sharon et al., 2019) and they have developed microbiome transfer therapy (MTT) and observed a reduction in ASD-related symptoms (Kang et al., 2017).

The intestines are also the largest immune organ, gathering more than 70% of the immune cells as a vital digestive organ. Gut microbiota is also related to the host’s immune system (Vatanen et al., 2016). Gut microbiota and metabolites can induce the production of helper T cells (Th) and regulator T cells (Tregs), which contribute to the maturation of host adaptive and innate immunity (Rooks and Garrett, 2016; Shi et al., 2017; Kayama et al., 2020). It can be inferred that autoimmune diseases are closely related to intestinal flora (Levy et al., 2017). There are several studies on the gut microbiota and metabolome among GD patients, and many results strongly support a role for the gut microbiota in GD and GO (Moshkelgosha et al., 2021).

### GD and Gut Microbiota

Some previous studies demonstrated the connections between the gut microbiome and AITD (Köhling et al., 2017). Many studies showed that GD is related to *yersinia* enterocolitica, e.g., mice fed only with *yersinia* enterocolitica did not develop GD (Weiss et al., 1983; Wang et al., 2010). There were also significant differences in the microbiota profile between HT patients and healthy controls (Zhao et al., 2018). Zhou et al. characterized the gut microbiota in hyperthyroid patients (Zhou et al., 2014). There is limited research on the relationships between Graves’ disease and the gut microbiome. However, thyroid hormone levels correlate with the gut microbiome and the diversity of gut bacteria in patients with GD (Ejtahed et al., 2020). Bacteroidetes and Firmicutes are dominant species in the human gut. The ratio of Firmicutes to Bacteroidetes is commonly considered a representative of health status (Chen et al., 2016; Indiani et al., 2018). In the disease state, these two phyla tend to show significant changes. For example, Jiang et al. showed that GD patients had reduced alpha diversity compared with healthy individuals. At the phylum level, GD patients had a significant higher proportion of Bacteroidetes and a significantly lower proportion of Firmicutes than the controls (Jiang et al., 2021). Ishaq et al. also demonstrated this phenomenon in their study (Ishaq et al., 2018). They found that the diversity of gut bacteria in GD patients was less diverse in terms of richness than in healthy people. The proportion of Firmicutes in GD was lower than that in the control group, while the proportion of Bacteroidetes was higher than in the control group (Ishaq et al., 2018). Interestingly, this finding is consistent with what was observed in obese patients. Previous studies have found that obese people tend to have more Firmicutes, while lean people tend to have more Bacteroidetes (Schwiertz et al., 2010b; Riva et al., 2017). Further research work is required about the effects of thyroid hormones on gut microbiota. Besides Firmicutes and Bacteroidetes, there were also significant changes in the ratios and abundances of other phyla. Yan et al. showed that the number of Lactobacillales, Bacilli, Megamonas, Prevotalla and Veillonella strains were increased among GD patients (Yan et al., 2020). However, the number of Rikenellaceae, Ruminococuus and Alistipes strains was decreased among GD patients. In addition, the diversity of gut flora was decreased in patients with GD (Yan et al., 2020). There were also significant changes in gut microbiota in GO patients. Shi et al. found that the bacterial diversity (Simpson and Shannon) was reduced in patients with GO compared to the controls. At the phylum levels, the proportion of Bacteroidetes increased and Firmicutes decreased significantly in GO than that in controls. There were obvious differences in bacterial profiles between the two groups (Shi et al., 2019a). Then, Shi et al. further explored the differences in the compositing of gut microbes between GO and GD patients (Shi et al., 2021). At the phylum levels, the proportion of Chloroflexi was decreased significantly in GO patients. At the genus levels, Bilophila and Subdoligranulum were increased (Shi et al., 2021). It is reported that there are three gut bacteria genera (*Bacteroides*, Prevotella, Alistipes) that could separate GD patients from healthy individuals with 85% accuracy (Su et al., 2020).

Thyrotropin receptor antibody (TRAb) is a characteristic indicator of GD, with sensitivity and specificity greater than 95% for GD diagnosis (Massart et al., 2001; Cooper, 2003). Shi et al. believed that TRAb was significantly correlated with different levels of gut microbiota. At the family level, the proportion of Succinivibrionaceae was positively correlated to TRAb. At the genus level, Subdoligranulum was positively related to TRAb. At the species level, Parabacteroides distasonis showed an opposite correlation with TRAb. Their studies also suggested that GD patients with positive TRAb showed an increased risk of developing GO (Shi et al., 2019a). Prevotella and *Bacteroides* are positively correlated with TRAb in GO patients (Shi et al., 2019b).

### Metabolomics in the Study of GD

**FIGURE 1:**
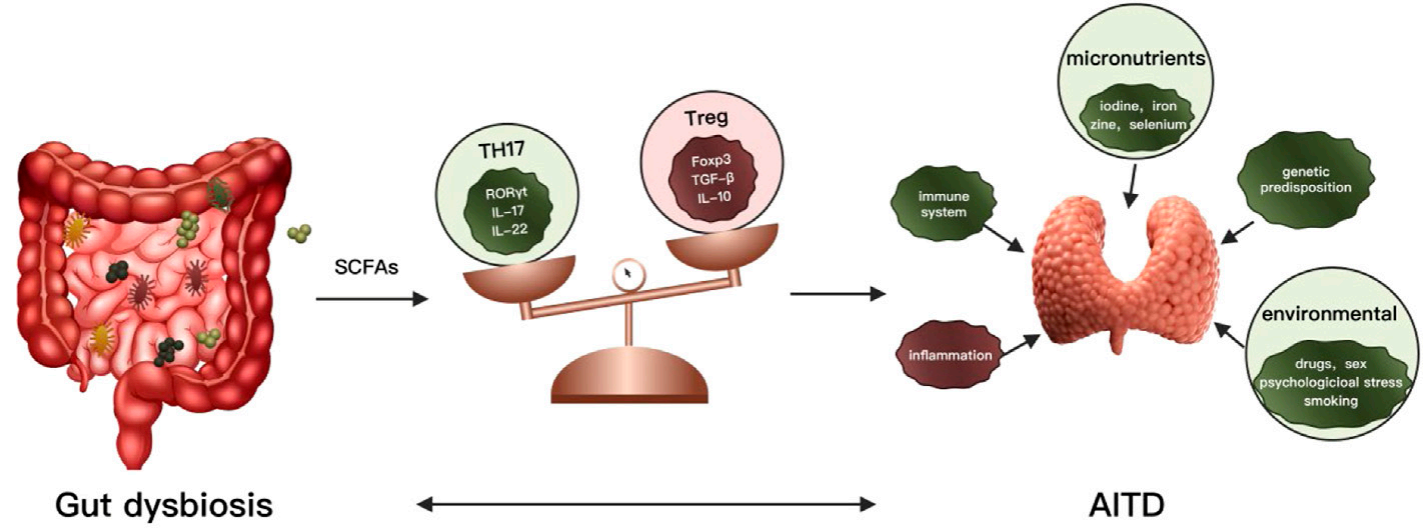
Association between gut microbiota, metabolites, and thyroid autoimmune diseases.

The dynamic balance of Th17 and Treg is closely related to the occurrence and development of various autoimmune diseases (Fasching et al., 2017). Treg cells are a subset of regulatory T cells that regulate the body’s autoimmune response. Tregs are characterized by the transcription factor Foxp3 (major regulators of Treg) and mainly exert immune suppressive effects. Maintaining immune homeostasis by secreting inhibitory factors (TGF-B, IL-10, IL-35) mediate immune suppressive effects by regulating TCR signaling promotes secretion and differentiation of anti-inflammatory cytokines (Göschl et al., 2019). The decrease of Treg cells increases the incidence and severity of AITD. And the number of Treg cells is significantly reduced in patients with GD (Saitoh and Nagayama, 2006; Nakano et al., 2007). The Th17 cells are also a subset of T helper cells by secreting interleukin 17 (IL-17, IL-22) induces inflammation and spread. IL-17 is involved in many inflammatory and autoimmune diseases, including systemic and organ-specific autoimmune diseases (Takeuchi et al., 2020; Yasuda et al., 2019). Th17 and IL-17 were increased in GD and participated in the development of GD. In patients with AITD, the proportion of Th17 cells in peripheral blood mononuclear cells (PBMCs) increased and higher mRNA level of their specific transcription factor RORγt in PBMCs (Li et al., 2016; Li et al., 2019). However, the level of Tregs and expression of Foxp3 mRNA were greatly decreased in AITD (Li et al., 2016; Li et al., 2019). Figueroa Vega et al. found that IL-17 was elevated in the thyroid tissues of GD RORγt mRNA patients, and both IL-17 and IL-22 levels were higher than healthy controls (Figueroa-Vega et al., 2010). Di. Peng observed that the concentration of IL-17 and IL-22 in plasma of GD patients was significantly higher than that of healthy controls, which was consistent with the increase of Th17 cells and positively correlated with TSAb (Peng et al., 2013). However, some studies have shown the opposite results (Yuan et al., 2017). The metabolites of the gut microbiome have been associated with the generation of proinflammatory cytokines and the production of Th17 cells. Commensal bacteria and their metabolites can also promote Treg generation and suppress the immune system (Haase et al., 2018). SCFAs are produced by the fermentation of non-digestible carbohydrates such as dietary fiber by gut bacteria, including butyrate (C (Shi et al., 2019a)), propionate (C (Ishaq et al., 2018)) and acetate (C (Bahn, 2003)), are essential metabolites in maintaining homeostasis (Luu and Visekruna, 2019). SCFAs have been proved to alter chemotaxis and phagocytosis, changes in cell function and proliferation, induction of reactive oxygen species (ROS), anti-tumor and anti-inflammatory (Tan et al., 2014). SCFAs contribute to the maintenance of intestinal barrier integrity and its regeneration effect on the intestinal epithelium (Memba et al., 2017). SCFAs are valuable sources of nutrients for enterocytes, together with thyroid hormones (chiefly triiodothyronine), stimulating enterocyte differentiation (Cayres et al., 2021; Meng et al., 1999). It also increases intercellular integrity and reduces the risk of a “leaky gut” by improving the adhesion of intestinal cells and reducing the PH in the intestinal tract, thus avoiding the invasion of pathological organisms (Memba et al., 2017; Bargiel et al., 2021). It is suggested that GD’s development is often linked to a compromised intestinal barrier (Knezevic et al., 2020). Recent studies emphasized the immunomodulatory potential of SCFAs in various autoimmune diseases and inflammatory disorders such as multiple sclerosis (MS), colitis, rheumatoid arthritis and AITD. The relation between SCFAs and thyroid function seems to be confirmed by several studies in the scientific literature describing changes in the gut microbiota, including concentrations of SCFAs in impaired thyroid status (Virili et al., 2018; Liu et al., 2020). Currently, two essential functions for SCFAs have been identified, inhibition of histone deacetylases (HDACs) and activation of G-protein coupled receptors (GPCRs), particularly GPR43, GPR41 and GPR109 A (Tan et al., 2014) (Sivaprakasam et al., 2016). Butyrate has been shown to have a positive effect on rheumatoid arthritis (Hui et al., 2019), inflammatory bowel disease (IBD) (Zhou et al., 2018) and autoimmune hepatitis (AIH) (Hu et al., 2018) by rebalancing between Treg and Th17 and increasing the number of Treg cells and decreasing Th17 cells in the system (Figure 1). Propionate is found to affect multiple sclerosis (MS) (Duscha et al., 2020) and GD (Su et al., 2020). However, little is known about the role of the SCFAs in Graves’ disease.

Struja et al. predicted the relapse of hyperthyroidism based on the assessment of metabonomics differences. Pyruvate and triglycerides are considered as predictors with AUCs of 0.73 and 0.67 (Struja et al., 2018). Al-Majdoub and others reported changes in the carnitine metabolism of GD patients prior to treatment compared to posttreatment (Al-Majdoub et al., 2017). The level of short-chain acylcarnitine decreased, medium-chain acylcarnitine increased, and long-chain acylcarnitine remained unchanged. The authors speculated that these phenomena reflect a starvation process that induced by hyperthyroidism (Al-Majdoub et al., 2017). Lipid profile from plasma and urine samples of GD patients was significantly different compared to controls. Some of Glycerophosphoethanolamine (PE), Glycerophosphoinositol (PI), Triacylglycerol (TG) and Glycerophosphoglycerol (PG) have changed significantly (Byeon et al., 2018). Polyamine metabolic profiles are also altered in AITD. GD and HT patients showed the same change relative to the control group for most of the polyamine metabolites. l-arginine (L-ARG), l-omithine (L-ORN), lysine (LYS) agmatine (AGM) are significantly and N-acetylputrescine (NPUT), spermine (SPM), 1,3-diaminopropane (DAP) are lower than the control group. However, GD and HT have different characteristics of change. GD patients had significantly lower cadverine (CAD) and higher N-acetylspermidine (NSPD), spermidine (SPD) and r-Aminobutyric (GABA) acid than the control group. But N-acetylspermine (NSPM) was decreased in HT. The anti-inflammatory effect of SPM was better than that of SDP. SPM/SPD can be more effective for estimating the anti-inflammatory effect. A decrease in SPM/SPD in patients with AITD indicated reduce in protective polyamines. SPM/SPD was negatively correlated with inflammatory chemokine IP-10 and TPOAb (Rider et al., 2007; Song et al., 2019). Ji et al. performed a non-targeted metabolomics analysis on the blood and orbital tissues of GD, GO and healthy controls. They identified ten differential metabolites in the disease group (gluconic acid, glucose, pelargonic acid, threose, fumaric acid, glycerol, mannose, pentade canoic acid, pyruvate, and 2- (4-hydroxyphenyl)ethanol) (Ji et al., 20188). The metabolite panel achieved an accuracy of 0.931 and the sensitivity and specificity are 0.787 and 0.875, respectively (Ji et al., 20188). Among the metabolite panel, almost all metabolites showed a positive correlation with the levels of TRAb (Ji et al., 20188). Propionate was significantly reduced in GD patients, which was negatively correlated with FT3, FT4, TRAb level, and positively correlated with TSH level (Su et al., 2020). At present, there are not many studies on GD metabolomics, and the specific association and mechanism still need to be further studied.

Gut dysbiosis can lead to changes in metabolites such as SCFAs. As a consequence, the balance of Th17 and Tregs would be damaged, leading to an autoimmune response and causing autoimmune thyroid diseases. AITD: autoimmune thyroid diseases; IL: interleukin; Th: T helper cell; Tregs: regulatory T cells.

### Microbiome and Metabolome in GD Study

In the last 20 years, it has been established that the gut microbiome plays an essential role in maintaining host health and the occurrence and progression of the disease. Metabolites are the primary way that gut microbes interact with hosts. The small molecules generated or modified from microorganisms can be detected in urine, serum, feces, cerebrospinal fluid, and other tissues (Holmes et al., 2011; Del Rio et al., 2017). The homeostasis of a healthy intestinal environment is regulated by the balance of microbiota, metabolites, and immune systems. In the state of disease, the intestinal balance is usually destroyed. Studies showed that gut dysbiosis leads to Treg/Th17 imbalance through the propionic acid regulation pathway, which, together with other pathogenic factors, promotes GD occurrence (Su et al., 2020). Gut dysbiosis was mainly manifested by a significant decrease in SCFAs-producing bacteria and SCFAs. *Bacteroides fragilis* YCH46 strain in GD patients was obviously reduced compared to healthy controls. It can produce propionic acid, increase the number of Treg cells and reduce the number of Th17 cells. Therefore, *B. fragilis* YCH46 was a natural activator of Treg cells and inhibitor of Th17 cells (Rios-Covian et al., 2015). YCH46 strain of *B. fragilis* provides a new direction for the treatment of GD. It can improve immune dysfunction in GD patients and be used as an immunomodulator or as an auxiliary treatment for GD patients to reduce recurrence rate (Su et al., 2020). A recent study found significant differences in metabolic pathways between GD groups and healthy controls. Formaldehyde assimilation and allantoin degradation, mevalonate and isoprene biosynthesis significantly increased in the GD patients. In contrast, the microbial metabolic abilities of fatty acid biosynthesis, pyruvate fermentation to hexanol, anaerobic energy metabolism, creatinine degradation and gluconeogenesis decreased significantly in relative abundance in the patients. The change of gut microbiota is Butyricimonas *faecalis*, Faecalibacterium prausnitzii, Akkermansia muciniphila and Bifidobacterium adolescentis decreased in the GD, whereas Veillonella parvula, Eggerthella lenta, *Fusobacterium* mortiferum, *Streptococcus* parasanguinis, and *Streptococcus* salivarius were enriched. And use propionic acid, acetic acid, cholate and chenodeoxycholate as potential biomarkers (Zhu et al., 2021). Jiang et al. found that Blautia, Eubacterium and Anaerostipes were decreased in GD. Eubacterium and Anaerostipes produce butyric acid and maintain the integrity of the intestinal epithelium as well as induce the generate of Treg cells to strengthen the tightness of the intestinal mucosal barrier (Duncan et al., 2004; Venkataraman et al., 2016; Jiang et al., 2021). The primary metabolite of Blautia is butyric acid and has been shown to have anti-inflammatory effects (Jenq et al., 2015). The decrease of these three butyric acid-producing bacteria leads to the reduction of butyric acid and inhibits the differentiation of Treg cells, resulting in immune system dysfunction and eventually the development of AITD (Jiang et al., 2021).

## Discussion

Autoimmune diseases are still challenging for the clinic. Changes in the composition and abundance of the gut microbiota, as well as related metabolites, are closely linked to the occurrence of GD. These findings provide some potential biomarkers for early diagnosis of GD, and some new probiotics related to GD can be used for adjunctive treatment and prevention of recurrence. However, related studies on gut microbiota metabolome in patients with GD are relatively lacking, and further studies are needed. It is believed that probiotics have positive effects on thyroid diseases, which has been confirmed *in vitro* cell studies and animal studies. However, these effects on human beings still require intensive investigations. Accurate qualitative-quantitative characterization of probiotics according to different pathological stages are also needed. Current metabolomics studies provide the correlations between gut micrbiota and the disease, however, the molecular mechanism between gut microbiota and GD remain unclear. One of the key point is how the metabolites synthesized by the gut microbiota. This is essential for the following development of related medicines.

The ultimate goal for the multi-omics study is to develop new diagnostic standards (microbial/metabolite biomarkers) and treatment strategies (probiotics/targeted microbial therapy or functional metabolites) for GD, with an individual treatment plan for each patient to achieve a complete cure and prevent a recurrence.
